# Insecticide resistance levels and mechanisms in *Aedes aegypti* populations in and around Ouagadougou, Burkina Faso

**DOI:** 10.1371/journal.pntd.0007439

**Published:** 2019-05-23

**Authors:** Athanase Badolo, Aboubacar Sombié, Patricia M. Pignatelli, Aboubakar Sanon, Félix Yaméogo, Dimitri W. Wangrawa, Antoine Sanon, Hirotaka Kanuka, Philip J. McCall, David Weetman

**Affiliations:** 1 Laboratoire d’Entomologie Fondamentale et Appliquée, Université Joseph Ki-Zerbo, Ouagadougou, Burkina Faso; 2 Department of Vector Biology, Liverpool School of Tropical Medicine, Liverpool, United Kingdom; 3 Université Norbert Zongo, Koudougou, Burkina Faso; 4 Center for Medical Entomology, The Jikei University School of Medicine, Tokyo, Japan; 5 Department of Tropical Medicine, The Jikei University School of Medicine, Tokyo, Japan; University of Veterinary Medicine, Vienna, AUSTRIA

## Abstract

**Background:**

Recent outbreaks of dengue and other *Aedes aegypti*-borne arboviruses highlight the importance of a rapid response for effective vector control. Data on insecticide resistance and underlying mechanisms are essential for outbreak preparedness, but are sparse in much of Africa. We investigated the levels and heterogeneity of insecticide resistance and mechanisms of *Ae*. *aegypti* from contrasting settings within and around Ouagadougou, Burkina Faso.

**Methodology/Principal findings:**

Bioassays were performed on larvae and adults to diagnose prevalence of resistance, and to assess levels where resistance was detected. Investigation of resistance mechanisms was performed using synergist bioassays, knockdown resistance (*kdr*) target site mutation genotyping and quantitative PCR expression analysis of candidate P450 genes.

Larval dose-response assays indicated susceptibility to the organophosphates tested. Adult females were also susceptible to organophosphates, but resistance to carbamates was suspected in urban and semi-urban localities. Females from all localities showed resistance to pyrethroids but resistance prevalence and level were higher in urban and especially in semi-urban areas, compared to the rural population. Environment was also associated with susceptibility: adults reared from larvae collected in tires from the semi-urban site were significantly less resistant to pyrethroids than those collected from large outdoor drinking water containers (‘drums’). Susceptibility to both pyrethroids tested was largely restored by pre-exposure to Piperonyl Butoxide (PBO), suggesting a strong metabolic basis to resistance.

The 1534C *kdr* mutation was nearly fixed in semi-urban and urban areas but was far less common in the rural area, where the 1016I *kdr* mutation frequency was also significantly lower. P450 gene analysis detected limited over-expression of single candidates but significantly elevated average expression in the semi-urban site compared to both a susceptible laboratory colony, and females from the other collection sites.

**Conclusions/Significance:**

Our results reveal pyrethroid resistance and paired *kdr* mutations in both urban and semi-urban sites at levels that are unprecedented for mainland Africa. The combination of target site and metabolic mechanisms is common in *Ae*. *aegypti* populations from other continents but is a worrying finding for African populations. However, organophosphate insecticides are still active against both larvae and adults of *Ae*. *aegypti*, providing useful insecticidal options for control and resistance management.

## Author summary

Several African countries including Burkina Faso have experienced dengue outbreaks recently. In outbreaks, dengue control relies on the control of its vector but data on *Aedes aegypti* resistance to insecticide, a key for efficient control, are often lacking, especially for the African continent. We conducted a study in localities within and around Ouagadougou to assess the *Ae*. *aegypti* resistance to insecticides and investigate the mechanisms involved. We collected larvae of *Aedes aegypti* from three different localities and different breeding sites to assess larval and adult susceptibility to insecticides. *Aedes aegypti* adults showed high resistance to pyrethroid insecticides with the prevalence and intensity of resistance depending on the locality and type of breeding site. Adults showed less pronounced resistance to carbamates, and both larvae and adults remain susceptible to organophosphate insecticides. The resistance to pyrethroid insecticides is partly explained by a high frequency of a pair of kdr mutations (1534C and 1016I) and the overexpression of genes of the P450 family linked to insecticide degradation in the mosquito. Datasets on both resistance and mechanisms in *Ae*. *aegypti* from the African continent are quite rare and are important for dengue control in Burkina Faso and elsewhere.

## Introduction

The African continent is particularly at risk of arbovirus-disease outbreaks, a situation enhanced by the high number of vector species, the lack of organised vector control, a deficit of vector biologists and the absence of prevention policies for neglected tropical diseases [1]. *Aedes aegypti* is the main vector involved in the transmission of the most important arboviruses—dengue, yellow fever, Zika and chikungunya—which occur as recurrent outbreaks in parts of the African continent [2]. Approximately 50% of the world’s population lives in areas at risk of dengue virus infection, and in Africa, dengue has been recorded in 34 countries in the past 50 years [3,4]. West Africa has been identified as a potential dengue hotspot because of the co-occurrence of rapid urbanization without adequate sanitation and the widespread presence of *Ae*. *aegypti* [5].

Burkina Faso has a long history of dengue epidemics, with the first reported in 1925 [3] and another in 1982 [6]. Dengue cases were recorded regularly but at low levels from 2006 [7,8] until an outbreak in 2016 resulted in 2,600 cases and 21 deaths [9]. In 2017, a larger outbreak in the city of Ouagadougou spread to other regions, ultimately resulting in 14,455 cases and 29 deaths nationwide [10]. These outbreaks highlighted the vulnerability of Burkina Faso to dengue and other *Aedes*-borne arboviral diseases.

Typically, dengue prevention relies on vector control through larval source reduction and case management, but in outbreak periods, insecticidal space spraying is usually employed to target adult mosquitoes [11]. Worldwide, dengue vectors have developed resistance to most insecticides used in public health [12] but data from Africa are sparse, and the mechanisms involved in resistance in African *Aedes* populations are very poorly understood [1].

The best documented mechanisms of *Ae*. *aegypti* insecticide resistance involve mutations in the voltage gated sodium channel (VGSC) target site of pyrethroids and Dichloro-Diphenyl-Trichloroethane (DDT), and metabolic detoxification. Multiple VGSC knockdown resistance (*kdr*) mutations have been identified in *Ae*. *aegypti* but only V410L, V1016G, I1011M and F1534C have been validated as being directly causally-associated with resistance to pyrethroid insecticides [13–15]—and only one of these, F1534C, has been detected in Africa to date [16]. Other mutations (e.g. V1016I and S989P) are involved in the resistance at least when associated with other VGSC variants [12]. Metabolic resistance is also important in *Ae*. *aegypti* populations at multiple geographic locations, and many genes of the P450 family, especially from the CYP9 and CYP6 subfamilies have been associated with resistance to pyrethroids [12,17].

*Aedes aegypti* is rarely a target for vector control in Africa, though in Cape Verde insecticide-based vector control has been established since 2009 after the first dengue cases were diagnosed [18]. Current data suggest a more heterogeneous picture of insecticide resistance across Africa than in Latin America and South-East Asia, but in mainland West Africa there is evidence of established or emerging resistance to DDT, carbamates and pyrethroids [1]. Knowledge of the underlying mechanisms is very limited in African populations, but resistance to DDT and permethrin has been linked to a high frequency of the 1534C *kdr* mutation in Ghana, whilst the 1016I mutation which, when co-occurring with 1534C yields broader and stronger pyrethroid resistance [19], was very rare [16]. This is in contrast to Cameroon, where resistance to pyrethroids has evolved within a decade from susceptibility to well-established resistance, apparently in the absence of *kdr* mutations [20,21]. On the island of Madeira, the *Ae*. *aegypti* population exhibit strong pyrethroid resistance, underpinned by dual *kdr* mutations (V1016I and F1534C) and overexpression of metabolic genes. Since the Madeira population was founded only recently, it suggests, worryingly, that a suite of resistance mechanisms can establish rapidly after introduction to an area [22].

Information on insecticide resistance is a basic requirement when considering tools or approaches for dengue control. In this study, we characterise the resistance of *Ae*. *aegypti* and investigate underlying mechanisms in urban, semi-urban and rural localities of Ouagadougou, the capital city of Burkina Faso. Key findings include the vector population’s susceptibility to organophosphates and strong but variable resistance to pyrethroids between localities, linked to the 1534C and 1016I *kdr* mutations and to P450 gene overexpression.

## Materials and methods

### Collection localities

**Fig 1:**
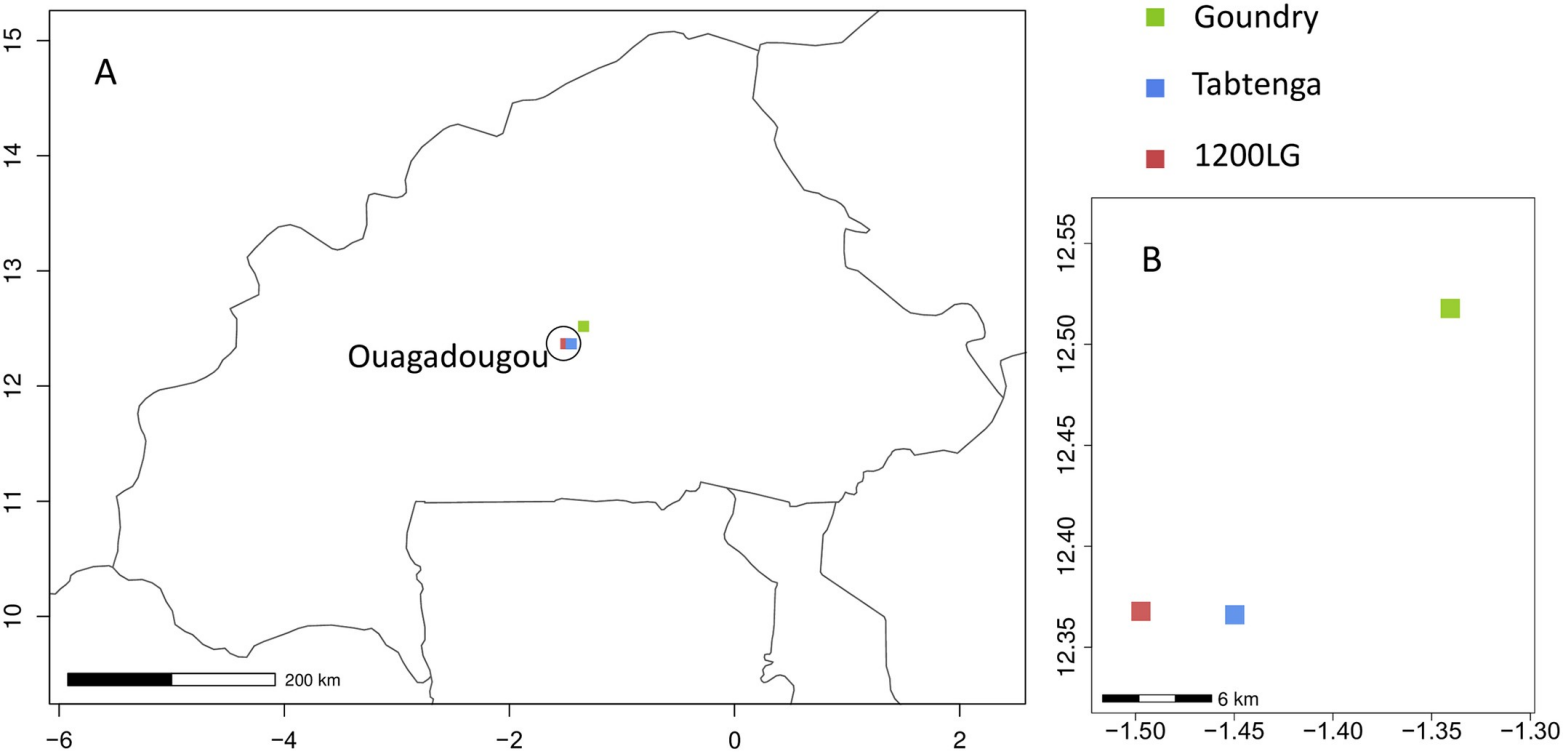
Map of the study localities. Fig 1A represents the map of Burkina Faso showing the capital city Ouagadougou (circled) and the study localities (colored squares). Fig 1B shows the relative location of the study sites, Goundry, Tabtenga and 1200 logements (1200LG). On both figures, longitude is on X axis and latitude is on Y axis.

*Aedes aegypti* larvae were sampled from two localities within, and one beyond the perimeter of the city of Ouagadougou (Fig 1), selected for differences in their ecological characteristics, human population size and housing type.

1200 Logements (12° 22' 3.569''N, 1° 29' 50.24''W): located within central Ouagadougou close to the international airport; housing has piped water supply with good sanitation and drainage, electricity and waste management systems. Potential *Aedes* breeding sites are predominantly discarded tires and small containers (volume <5L).

Tabtenga, (12° 21' 58.039''N, 1° 26' 59.074''W) located approximately 5 km East of 1200 Logements, Tabtenga is a semi-urban district, lacking a centralized water supply, electricity or waste management systems. Potential *Aedes* breeding sites include tires, drums (large ceramic water containers) and small containers.

Goundry (12°31´4.262”N, 1°20´25.771”W): a small rural farming community situated 25 km north-east of Ouagadougou; mostly small scale cultivation and livestock, with no water supply, electricity or waste management systems. Potential *Aedes* breeding sites are primarily drums, or water containers provided for animals.

### Larval collection and laboratory rearing

During the rainy season from August to October 2016, larvae were collected in tires from 1200 Logements, in drums and tires from Tabtenga, and in drums from Goundry. Water containing larvae from breeding sites was filtered using sieves and the larvae transferred to the laboratory. Many breeding sites were sampled from each locality and larvae were pooled for subsequent rearing. Larvae were reared using dried cat food in the insectary until F0 adult mosquitoes of three to five days old were obtained for bioassay tests. The insectary conditions were 27.7±1.4°C temperature, 79.1±5.5% relative humidity, 12h light/dark photoperiod.

### Larval susceptibility tests

Larval bioassays were performed according to WHO dose-response assay protocols [23] on 3^rd^ and 4^th^ instar larvae after one day of acclimation to the laboratory conditions as described above. Three organophosphate insecticides were tested, at five concentrations for each insecticide: temephos (0.25 mg/L to 20.25 mg/L); fenitrothion (0.25 mg/L to 20.25mg/L) and malathion (6.25 mg/L to 168.75 mg/L). One hundred *Ae*. *aegypti* larvae were exposed to each concentration, along with a negative control (no insecticide); numbers of dead and alive larvae were recorded at the end of the 24 h exposure period. We were unable to obtain a laboratory susceptible strain as a standard on which to simultaneously perform bioassays locally. Therefore, to compare with values estimated from the data (see below) we obtained LC_50_ values (in mg/L) from published literature to allow computation of resistance ratios. Moyes et al. [12] reviewed published data on temephos larval assays using the *Ae*. *aegypti* Rockefeller strain, and calculated a mean LC_50_ = 0.0042 (N = 30). We found three studies that estimated malathion LC_50_ values for susceptible strains: Rockefeller = 0.27 [24]; Rockefeller = 0.40 [25]; GA1 strain = 0.097 [26], and took the median of these values (0.27 mg/L) as the reference LC_50_. For fenitrothion we identified only one study with the Rockefeller strain (0.009 mg/L; [25], which we used as our reference value.

### Adult susceptibility tests

Adult bioassays used females (three to five days post-eclosion) reared from field-collected larvae and were performed according to standard protocols [27]. Five insecticides were tested: 0.75% permethrin; 0.05% deltamethrin; 0.1% bendiocarb; 1% fenitrothion and 5% malathion; and the resistance status of each population interpreted according to WHO criteria [27]. Whilst these are not all accepted diagnostic doses for *Ae*. *aegypti*, they are the most commonly used [12]. One hundred adult mosquitoes from each locality were exposed to each insecticide, along with 50 mosquitoes exposed to control (no insecticide) papers. Immediately following 1h exposure, knockdown was recorded, and mortality recorded after 24h. Mosquitoes were stored over silica gel at -20°C for later DNA analysis, and samples of survivors and control were kept in RNAlater at -20°C for gene expression analysis.

The CDC bottle assay technique was used also to test for higher levels of resistance to those insecticides for which resistance was detected in the WHO assays. For this purpose, a prolonged exposure time of 2h was employed with knockdown recorded at the end of the exposure period and mortality recorded 24h later. Technical grade insecticides (>90% purity; Sigma-Aldrich) were used at discriminating concentrations [28]. Stock solutions were prepared at concentrations of 12.5 μg/ml for bendiocarb, 10 μg/ml for deltamethrin, 50 μg/ml for fenitrothion and 15 μg/ml for permethrin. For each insecticide, 1 ml was used to coat the 250 ml bottles, which were dried and kept in a fridge until use. In addition, the synergist piperonyl butoxide (PBO), which primarily blocks activity of P450s and some esterases [29], was used to assess a possible role for metabolic resistance mechanisms, at the recommended concentration of 400 μg/ml [28] in pre-exposure assays, followed by pyrethroid exposure.

### Detection of *kdr* mutations

DNA was extracted from two legs of each mosquito, which were transferred using clean forceps to PCR plate wells. The wells were sealed and the plate briefly centrifuged to ensure the legs were at the bottom of the well. Twenty μl of STE buffer (0.1M NaCl, 10 mM TrisHCl pH = 8.0, 1mM EDTA pH8.0) was added to each well containing the mosquito legs. The plates were heated at 95°C for 90 min and briefly spun again. The resultant extracts were stored at -20°C until use. The V1016I, F1534C, S989P and V1016G VGSC mutations were genotyped using the Taqman qPCR method [30]. Reactions were performed in 96 well plates by adding 5 μl of Taqman gene expression SensiMix (Applied Biosystem, Foster city, USA), 0.125 μl of primer/probe, 3.875 μl of molecular grade sterile water and 1 μl of the DNA extract. Reactions were run on an Agilent MX3000P qPCR thermal cycler using cycling conditions of an initial denaturation of 10 min at 95°C, followed by 40 cycles of 92°C for 15 min and 60°C for 1 min.

### Candidate gene expression analysis

Control mosquitoes from bioassays preserved in RNAlater were used for RNA extraction and cDNA preparation. Mosquitoes were washed in distilled water and pooled in batches of five per tube for RNA extraction using the PicoPure RNA Isolation Kit (ThermoFisher) according to manufacturer’s instructions. Quantity and quality of RNA was checked using a NanoDrop spectrophotometer and was kept at -80°C until further use.

Complementary DNA (cDNA) was synthetized using reverse transcriptase with Oligo(dt)20 primer according to manufacturer’s instructions.

Candidate genes for expression analysis were chosen based on previous implication of involvement in metabolic resistance [12,17]. All primer sequences and their origins are shown in S1 and S2 Tables. Standard curve analyses were performed for each primer pair to check the specificity and efficiency of amplifications. Seven cytochrome P450 candidate genes were chosen for analysis, along with two normalising genes (S1 Table). Real-time quantitative PCR reactions were performed in a total volume of 20 μl (7.8 μl DDW+10 μl SYBRgreen, 0.6 μl of each primer and 1 μl of cDNA) under the following conditions: 95°C for 3 min, followed by 40 cycles of 95°C for 10 s and 60°C for 10 sec. The relative expression level and fold change (FC) of each candidate gene relative to the susceptible Rockefeller strain was calculated using the ΔΔcT method [31].

### Data analysis

Larval 50% and 95% lethal concentrations and their confidence limits were calculated by fitting a logistic regression to mortality after 24h, using an R script for analysis of bioassays and probit graphs [32]. To interpret results in terms of susceptibility, we calculated resistance ratios compared to susceptible strain values, as described above, and interpreted resistance ratios as follows: <5, little resistance; 5–10, moderate resistance; >10, substantial resistance [33]. Adult bioassay data were analysed according to WHO criteria [27]: a population is considered resistant if the mortality after 24h is less than 90% and susceptible when the mortality is over 98%. Between the two values, the population is considered suspected to be resistant and confirmation is needed.

A generalized linear model (GLiM) was fitted to the 2h CDC bottles bioassay mortality for pyrethroid insecticides. The model initially included pyrethroid insecticide type, container type, pre-exposure to PBO, and all interactions, with terms removed sequentially until the minimal model was obtained. Gene expression data (ΔcT) values were compared between each locale and the Rockefeller strain using *t*-tests, following checks using F-tests for homogeneity of variances. Variation in fold change (calculated as 2^-ΔΔcT^, relative to the average of Rockefeller) among collection locations was tested using multivariate analysis of variance (MANOVA) for all genes, following checks for normality (Kolmogorov-Smirnov test) and homogeneity of variances (Levene’s test), and ANOVA for individual genes with Tukey’s test for pairwise comparisons. All tests were performed using SPSS v 23. Allele frequencies were compared among localities using χ^2^ tests.

### Ethics statement

The research protocol entitled (16–030) “Dengue in Burkina Faso: establishing a vector biology evidence base for risk assessment and vector control strategies for an emerging disease” (16–030) received ethical approval from the National Ethical Committee for Medical Research, Ministry of Health in Burkina Faso (Deliberation N°2016-6-073) on 6^th^ June 2016 and the Research Ethics Committee at the Liverpool School of Tropical Medicine on 15th July 2016. When larvae were collected inside or near a residence, permission (signed consent) from the owners/residents was obtained before entering their property or land.

## Results

### Insecticide susceptibility bioassays

Effect of exposure to malathion, fenitrothion and temephos on *Ae*. *aegypti* larvae collected from urban, semi-urban and rural localities of Ouagadougou are shown in Table 1. There were significant differences in the estimates obtained for both LC_50_ values and LC_95_ among the localities, although the rank order of these was inconsistent across the three insecticides. Importantly, in all cases the resistance ratios calculated from the LC_50_ values were low, and in no case did they indicate any evidence of significant resistance.

**Table 1:** Summary of dose-response values from larval bioassays (LC_50_ and LC_95_), mg/L, with 95% confidence limits (95%CL).

Insecticide	Locality	LC_50_	(95%CL)	LC_95_	(95%CL)	RR_50_
Malathion	Goundry	0.1210^a^	(0.0702–0.2053)	0.4065 ^a^	(0.2305–2.0925)	0.45
	1200LG	0.0792 ^a^	(0.0507–0.1176)	0.4465 ^a^	(0.2591–1.2836)	0.29
	Tabtenga	0.0722 ^a^	(0.0507–0.1176)	0.2762 ^a^	(0.1924–0.5070)	0.27
Fenitrothion	Goundry	0.0023 ^a^	(0.0012–0.0038)	0.0105 ^a^	(0.0057–0.0551)	0.21
	1200LG	0.0118 ^b^	(0.0089–0.0156)	0.0362 ^b^	(0.0252–0.0693)	1.23
	Tabtenga	0.0052 ^c^	(0.0036–0.0071)	0.0278 ^c^	(0.0182–0.0561)	0.57
Temephos	Goundry	0.0018 ^a^	(0.0009–0.0033)	0.0042 ^a^	(0.0026–0.0516)	0.43
	1200LG	0.0045 ^b^	(0.0029–0.0068)	0.0236 ^b^	(0.0138–0.0684)	1.07
	Tabtenga	0.0038 ^b^	(0.0028–0.0050)	0.0177 ^b^	(0.0123–0.0322)	0.90

Resistance ratios (RR_50_) were calculated by comparison with published values for the Rockefeller susceptible strain (see Methods). Different superscript letters (a,b,c) within a column highlight values that are significantly different based on non-overlapping confidence limits.

**Fig 2:**
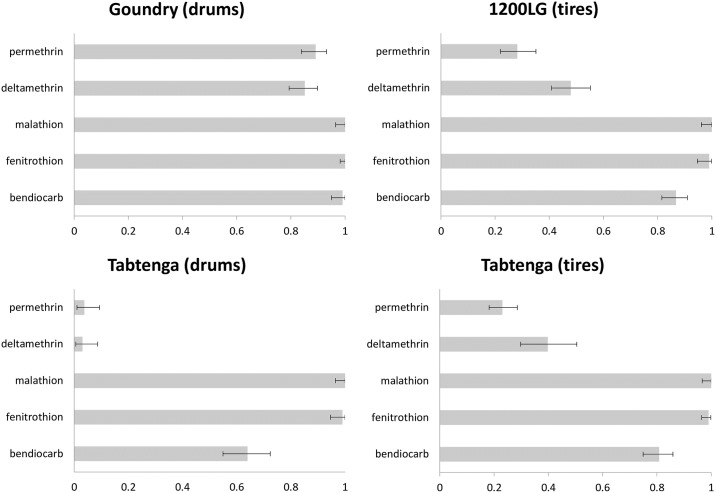
WHO 1h tube bioassay mortality (x-axis) from each site, with breeding site type indicated. Error bars show 95% binomial confidence intervals.

**Fig 3:**
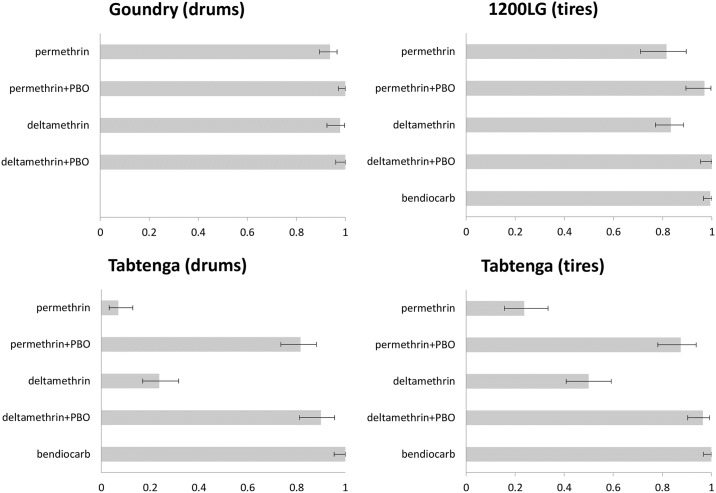
CDC 2h bottle bioassay mortality (x-axis) from each site, with breeding site type indicated. Error bars show 95% binomial confidence intervals.

Adult mosquito bioassay results are shown in Fig 2 for the WHO tube bioassays and Fig 3 for the CDC bottle bioassays. As there was no evidence of resistance to the organophosphate insecticides malathion and fenitrothion in any of the collection localities using the WHO bioassays, CDC assays were not performed for these insecticides. WHO test results for bendiocarb differed between collections (χ^2^_3_ = 53.4, P<<0.001) with Goundry susceptible, but 1200 Logements (1200LG) and Tabtenga resistant. However, in the Tabtenga collections, mosquitoes sampled from drums were significantly more resistant than those from tires (χ^2^_1_ = 10.9, P<0.001), while tire-collected mosquitoes showed similar bendiocarb bioassay results to those collected from 1200LG (χ^2^_1_ = 2.4, P = 0.12). No evidence of higher-level bendiocarb resistance was detected in either site in the CDC bottle bioassays (Fig 3).

For both pyrethroids, WHO bioassays showed confirmed resistance (i.e. <90% mortality) in each collection but with significant variation in prevalence (permethrin: χ^2^_3_ = 298.9, P<<0.001; deltamethrin: χ^2^_3_ = 186.4, P<<0.001). In each case, mortality was by far the highest in Goundry and lowest in the Tabtenga drum collections (Fig 3), but there were no significant differences between WHO bioassay results for the tire-collected mosquitoes from Tabtenga and 1200LG (permethrin: χ^2^_1_ = 1.3, P = 0.25; deltamethrin: χ^2^_1_ = 1.4, P = 0.24). Results from the 2h CDC assays indicated some reduction in susceptibility in Goundry, with mortalities >90% but <98% for permethrin and deltamethrin, respectively. In both cases, PBO pre-exposure restored full susceptibility (Fig 3). Mortality was lower in the 1200LG females (and largely restored by PBO) and lower still in those from both container types from Tabtenga (Fig 3; non-overlapping confidence limits indicate significance). In the Tabtenga collections, insecticide type, container and PBO all exerted highly significant influences on mortality, but the lack of any significant model interaction terms (Table 2), suggests independent effects, i.e. the differences between insecticides and the effects of PBO synergism were similar across container types.

**Table 2:** Generalized linear model analysis of 2h pyrethroid insecticide exposure; CDC bottle bioassay data for collections from Tabtenga.

Source	Wald χ^2^_1_	P-value	Mortality difference
Intercept	33.6	<<0.001	
Container	31.2	<<0.001	tire > drum
Insecticide	33.1	<<0.001	deltamethrin > permethrin
PBO	246.2	<<0.001	PBO > no PBO

The model includes container type, pyrethroid insecticide type and pre-exposure to a synergist as factor. All interaction terms were included initially but were non-significant and the minimal model is shown

### Molecular analysis of resistance mechanisms

**Fig 4:**
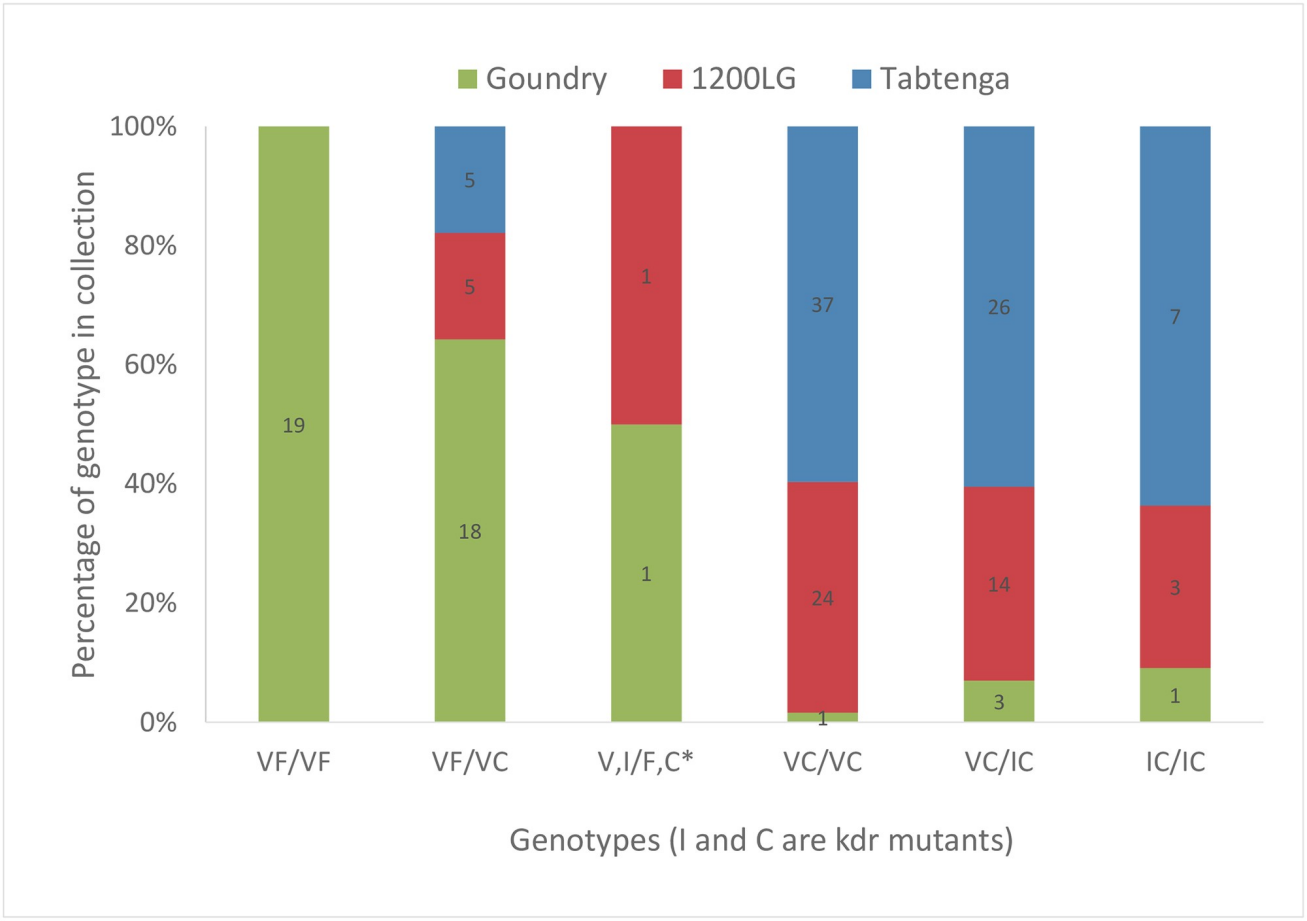
Genotype frequencies at the VGSC mutation positions V1016I and F1534C in females from each collection location. Inset numbers show the number of genotypes detected. VF/VF is wild type and VF/VC contains only one mutant allele; all other genotypes contain at least two mutant alleles. *heterozygote at each position, therefore genotypes could not be determined.

The target site mutations *kdr* S989P, V1016G, F1534C, and V1016I were genotyped in 48, 75, and 43 *Ae*. *aegypti* females from 1200LG, Tabtenga and Goundry, respectively (S5 Table). Only the V1016I and F1534C mutations were detected. The 1534C mutation was almost fixed in the urban (1200LG) and the semi-urban (Tabtenga) localities (which are separated by about 5 km) with allele frequencies of 0.94 and 0.97, respectively, but was far less common in rural Goundry (separated by about 25 km from the other localities), with an allele frequency of 0.34 (χ^2^_2_ = 149.8, P<<0.001). The V1016I *kdr* mutation was less common in all collection sites, but again it was found at higher frequencies in 1200LG (0.22) and Tabtenga (0.27) than in Goundry (0.06) (χ^2^_1_ = 18.6, P<0.001). Similarly, the frequencies of combined genotypes differed markedly (Fig 4) with the dual wild type genotype absent from 1200LG and Tabtenga but common in Goundry. Assuming that a single allele (across the two loci) is unlikely to exert much influence on resistance phenotype [34], we compared the frequencies of genotypes with zero or one mutant alleles with those with two or more mutations across populations (Fig 4). Frequencies differed dramatically (χ^2^_1_ = 48.3, P<0.001) as a result of the Goundry genotypes, with no significant differences (χ^2^_1_ = 0.19, P = 0.66) between 1200LG and Tabtenga.

**Fig 5:**
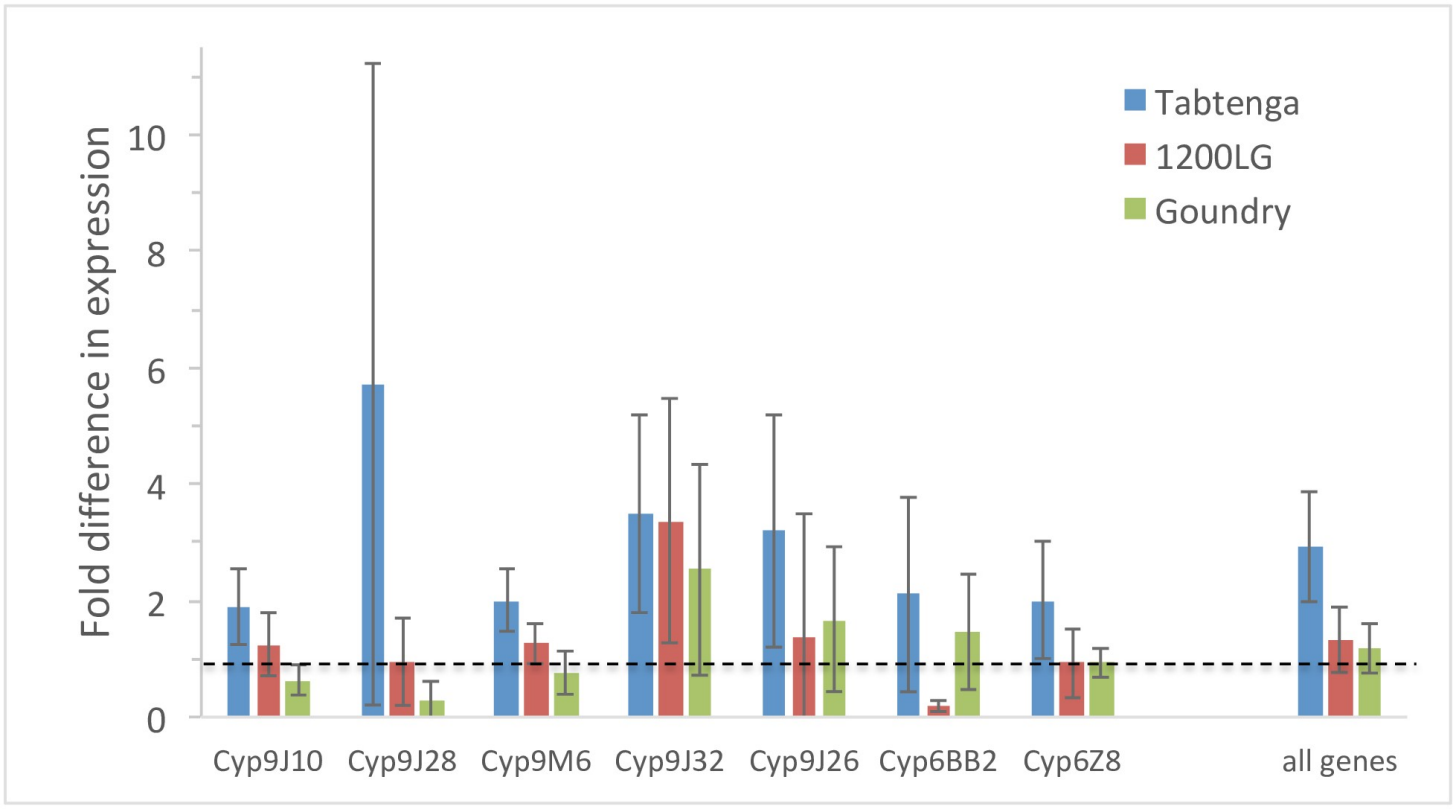
Expression of candidate P450 genes in relation to the susceptible Rockefeller laboratory strain, against which the dashed line indicates parity. Bars show mean fold change ± 95% confidence intervals. The final bars show the average expression across all genes.

Expression of Cyp6 and Cyp9 subfamily P450 candidate genes was analysed by comparing insecticide-unexposed mosquito samples from each locality relative to the Rockefeller susceptible strain. The P450 genes were generally expressed at low-moderate levels relative to Rockefeller and whilst expression was highest in Tabtenga females for every gene (Fig 5), no significant differences in expression from Rockefeller or among collections were detected for any individual gene after correction for multiple testing (minimum uncorrected P = 0.02). However, comparing fold differences (relative to Rockefeller) of the P450 genes jointly revealed significant variation among the three collection locations (MANOVA, Hotelling’s T^2^_12,14_ = 7.3, P = 0.027), with higher average expression for the Tabtenga collections than 1200LG or Goundry, neither of which differed from Rockefeller (Fig 5). This suggests that whilst none of the candidate P450 genes showed strong variation, their aggregate expression level may contribute to the variation in resistance phenotypes observed.

## Discussion

Resistance to insecticides in *Aedes* vectors of arboviruses is a major challenge for disease control globally. Here we investigated the susceptibility to commonly-used larval and adult insecticides in three contrasting localities of Ouagadougou, Burkina Faso, to provide essential information to aid rational insecticide choices for preventative control and dengue outbreaks. Unfortunately, a severe outbreak of dengue started before the end of our investigation, but our preliminary data showing *Ae*. *aegypti* susceptibility to malathion supported its use for outdoor spraying in hotspots during the outbreak. We also investigated the resistance mechanisms that may be involved in resistance, with particular relevance to pyrethroids. Overall, our study recorded no resistance to organophosphates, moderate and spatially-variable resistance to carbamates, and strong but highly-variable resistance to pyrethroids, likely driven by dual *kdr* mutations and metabolic resistance.

### Variable susceptibility to insecticide

*Aedes aegypti* control commonly employs organophosphates, particularly temephos and malathion, to control larval and adult stages, respectively. The LC_50_ values we obtained for temephos are very similar to values obtained for the Rockefeller susceptible strain [12] and whilst fewer published data are available for fenitrothion and malathion as larvicides, our data are compatible with a fully susceptible phenotype. These results reflect the overall picture from studies in Africa where, to date, temephos resistance appears absent [1], in contrast to the situation in Asia and especially Latin America [12]. We did not test other common larvicides such as *Bacillus thuringiensis* var *israeliensis* (*Bti*) or pyriproxyfen in this study, but there are currently no reports of resistance to either in *Ae*. *aegypti* [12], suggesting that multiple options for larval control exist in Burkina Faso, and likely elsewhere in mainland Africa.

In different parts of the world with longer histories of dengue control, *Ae*. *aegypti* populations are resistant to organophosphates [35–37], though inconsistencies in the diagnostic doses applied limit comparability [12]. In fact, the dose we applied for malathion is correct for *Anopheles*, but five-fold higher than that recommended for *Ae*. *aegypti* [27], although the recommended dose is very seldom applied [12]. Nevertheless, the dose used for fenitrothion is recommended for both *Aedes* and *Anopheles* [27], and, since full susceptibility was recorded for both insecticides, a conclusion of no resistance to organophosphates seems reasonable. Though uncommon, there are a few reports of relatively low-prevalence resistance to organophosphate adulticides from elsewhere in Africa [1]. Therefore, more detailed investigation of variation in the susceptibility profiles in adult female *Ae*. *aegypti* to malathion is recommended using the more informative dose-response methodologies. At present though, susceptibility to organophosphates in the localities we surveyed, coupled with the general rarity of organophosphate resistance in Africa, suggests that insecticides from this class are viable options for outbreak control. In contrast, we detected WHO-defined resistance to a carbamate, bendiocarb, in the urban (1200LG) and semi-urban (Tabtenga) sites, though not in the rural site (Goundry). Bendiocarb is less commonly used for *Aedes* control than organophosphates and pyrethroids, but a recent trial in Mexico found that bendiocarb was much more effective than deltamethrin for indoor residual spraying against a pyrethroid resistant population [38]. Full mortality in the 2h CDC bottle bioassays suggests that bendiocarb resistance is not at a high level in our survey sites, but it would still appear to be a less favourable option for adult control than organophosphates at present in Burkina Faso.

Populations from the urban and semi-urban areas showed moderate to very high resistance to both of the pyrethroid insecticides tested, and though at much lower prevalence, resistance was also detected to permethrin and deltamethrin in the rural site. From the WHO assays it was unclear whether resistance might differ between Tabtenga and 1200LG because results were almost identical when comparing adult females raised from collections from tires, but much lower mortalities were found in the collections from drums in Tabtenga. The longer duration CDC assays resolved this uncertainty, with significantly lower mortality in the Tabtenga than the 1200LG tire collections. Yet, in both the WHO and CDC assays, the Tabtenga drum collections showed significantly lower mortality than those from tires. The cause of this difference is unclear but seems most likely to be environmental, perhaps related to poorer developmental conditions in tires (e.g. lower food availability in these shaded habitats) or toxins leaching from the tires, although these have previously been linked to induction of P450s and potentially increased resistance in *Ae*. *albopictus* [39,40]. We are not aware of any previous demonstrations of such an effect of natural environmental variation on *Ae*. *aegypti* resistance, but given variation in the frequency of types of breeding sites found among areas [41], this could have an important impact on local resistance and deserves further investigation.

Pyrethroid resistance is found worldwide in *Ae*. *aegypti* [12], though the prevalence and higher-level resistance we detected in Tabtenga appears to be as strong as any yet reported from Africa [1]. The source of selection that may have driven resistance to this level is unclear. There is no history of targeted vector control for *Ae*. *aegypti* using pyrethroid insecticides in Burkina Faso, and the nature of the breeding sites means that run-off from agricultural application is a far less likely selective pressure than for *Anopheles* [42]. Increased insecticide pressure from malaria control interventions, is frequently linked to rising pyrethroid resistance in *Anopheles* [43–45]. Indeed in Goundry, *Anopheles gambiae* pyrethroid resistance and *kdr* mutation frequency increased between 2008 to 2011, which has been attributed to successive bednet distribution campaigns in Burkina Faso [46]. These may also have affected *Ae*. *aegypti* pyrethroid resistance in the three localities, although domestic use of insecticides may also constitute an important source of selection [47,48], especially in more affluent urban and semi-urban localities. Further work to identify sources of selection is clearly required if resistance management programs are to be a successful part of *Aedes* control programs.

### Pyrethroid resistance mechanisms

We genotyped four possible *kdr* mutant positions in our survey. The S989P and V1016G mutants which are important for pyrethroid resistance in Asia [12], and also Saudi Arabia [30] were absent in our study site. The 1534C *kdr* mutation is common in *Ae*. *aegypti* and has a worldwide distribution [12]. We found this mutation to be almost fixed in the urban and semi-urban localities, though far less common in the rural area. This mutation is known to occur in neighboring Ghana [16] though the highest allele frequency reported there (60%) is much lower than in Tabtenga or 1200LG. Similarly, the V1016I mutation, which was only detected in a single individual in Ghana, [16] is much more common in the localities we surveyed (>20%), and again was significantly less common in Goundry, than in 1200LG and Tabtenga. Excluding Goundry, the frequencies of these mutations in our sites confirm results from recent (2017) collections in another urban area of Ouagadougou [34], and are very similar to those detected on the island of Madeira, where the *Ae*. *aegypti* population is thought to have been very recently introduced [22]. Apart from Ghana and the recent data from Burkina Faso, there are few other results from mainland Africa, though in central Africa, neither *kdr* mutation has yet been found, despite established pyrethroid resistance [21].

In South and Central America [49–51], and in the Caribbean [37] the co-occurrence of the 1534C and 1016I mutations is common and usually present as either (1) 1014V/1534C or (2) 1014I/1534C. Both of these haplotypes can confer pyrethroid resistance when present as a homozygote for haplotype 1, a heterozygote of haplotypes 1 and 2, and especially a homozygote of haplotype 2 [19]. In Goundry, these mutant genotypes comprised only 14% of the sample, but in the other sites the combined mutant genotype frequency was around 90%, although double mutant homozygotes were rare (<10%). The much higher frequencies of resistant genotypes in 1200LG and Tabtenga than Goundry are likely to explain a significant portion of the difference in permethrin and deltamethrin resistance.

Pre-exposure to PBO restored a substantial part of the susceptibility to permethrin and deltamethrin, most noticeably in Tabtenga where the resistance level (as measured in the 2h CDC assays) was highest. Although enhanced insecticide penetration may also be involved, the PBO result suggests the involvement of metabolic resistance mechanisms involving P450s, and perhaps esterases [52]. Metabolic resistance in *Ae*. *aegypti* mediated by P450s appears very common [12,17], and whilst several genes are frequently implicated in resistance, some of which are proven pyrethroid-metabolizers, the role of specific genes remains unclear [12]. We examined seven candidate P450 genes, most of which have been shown to metabolize pyrethroids, and including four from the geographically-closest population (Madeira) from which transcriptomic data have been obtained [22]. Whilst individually we did not detect strong or significant overexpression of individual genes, a trend of stronger overexpression was evident in the Tabtenga collection, which overall was significantly greater than the susceptible Rockefeller strain or the other two collection sites. Some of these genes may play a role in the metabolic resistance phenotype suggested by the strong action of PBO, which underpins variation between Tabtenga and 1200LG, but perhaps more as an aggregate overexpression than one dependent on specific genes. Alternatively, it is possible that the P450 genes probably important for resistance elsewhere, may be less so in African mainland populations and we did not assay the most important genes for metabolic resistance. Transcriptomic studies of *Ae*. *aegypti* from Africa will be required to help resolve this uncertainty.

### Conclusion

Our results show a variable but alarmingly high level of pyrethroid resistance, underpinned by dual *kdr* mutations and metabolic resistance, perhaps involving some of the P450 genes we screened. Operational consequences of this resistance are unknown, but the use of pyrethroids for spraying as an outbreak control method would now appear to be unlikely to have significant impact. Moreover, with the source of selection unknown, return to susceptibility seems improbable. In contrast, susceptibility to both larvicidal and adulticidal organophosphates indicates that effective options for control still exist. Additional insecticide classes, and non-insecticidal interventions, should also be tested in order to operate a successful resistance management programme. Typically, insecticide resistance is considered a highly heritable trait, but our results also highlight how differences in breeding habitats can exert a strong influence on resistance. This phenomenon has been little-investigated and warrants further research.

## Supporting information

S1 Table Sequences of primers used for qRT-PCR. (DOCX)

S2 Table WHO tubes bioassay data. (DOCX)

S3 Table CDC bottles bioassay data. (DOCX)

S4 Table Gene expression qPCR data. (DOCX)

S5 Table Genotyping data. (DOCX)
