# Supplementary material for: Insecticide resistance levels and mechanisms in Aedes aegypti populations in and around Ouagadougou, Burkina Faso
Source: PLoS Negl Trop Dis. 2019 May 23;13(5):e0007439. doi: 10.1371/journal.pntd.0007439 (PMC6550433; doi:10.1371/journal.pntd.0007439)
Supplement: S1 Table — (DOCX) [file pntd.0007439.s001.docx]

| **Table S1**: Spreadsheet of primers sequences used for quantitative Real Time –PCR (qRT-PCR) | | | | | | | |  |
| --- | --- | --- | --- | --- | --- | --- | --- | --- |
|  |  |  |  |  |  |  |  |  |
| **Gene name** | **accession** | **F primer** | **R primer** | **F primer sequence** | **R primer sequence** | **efficiency** | **R2** | **source of primers** |
| Cyp9J10 | AAEL006798 | cyp9j10F | cyp9j10R | ATCGGTGTTGGTGAAAGTTCTGT | CATGTCGTTGCGCATTATCCC | 103,5 | 0,993 | Bariami et al. PLOS NTDs 2012 |
| Cyp9J28 | AAEL014617 | cyp9j28aF | cyp9j28aR | CCACTGACGTACGATGCGA | GCCGATCAGTGGACGGAGC | 92,4 | 0,998 | Bariami et al. PLOS NTDs 2012 |
| Cyp9M6 | AAEL001312 | 9M6F88 | 9M6R89 | TCGGTGCACAATCCAAACAAC | GTCGGGTACGACCAACGAAA | 103,8 | 0,999 | Kasai et al. PLOS NTDs 2014 |
| Cyp9J32 | AAEL008846 | CYP9J32F | CYP9J32R | CGGTCCGCTTATGACGAAGAG | TTTGTTCGCTCCGAAGAGTGG | 102 | 0,993 | Seixas et al. PLOS NTDs 2017 |
| Cyp9J26 | AAEL014609 | cyp9j26aF | cyp9j26aR | CCTCTCCTGCTGCGAAAGGTC | CGTCTGAACATCCCGAAAACTTT | 105,6 | 0,997 | Bariami et al. PLOS NTDs 2012 |
| Cyp6BB2 | AAEL014893 | 6BB2F3q | 6BB2R4q | AGAAAGAGCACAGCTGCGAAA | ACTGCCGGCTGGAAGAAGTT | 103,6 | 0,998 | Kasai et al. PLOS NTDs 2014 |
| Cyp6Z8 | AAEL009131 | 6Z8F8q | 6Z8R9q | CGCGAATTGGTGTCACGATG | TGAACGCATCGTTCGGATCATTAA | 99,9 | 0,997 | Kasai et al. PLOS NTDs 2014 |
| RPS3 | AAEL008192 | aegRPF7 | aegRPR8 | AGCGTGCCAAGTCGATGAA | GTGGCCGTGTCGACGTACT | 102,8 | 0,997 | Kasai et al. PLOS NTDs 2014 |
| Ae60s L8 | AAEL000987 | Ae60sL8F | Ae60sL8R | CTGAAGGGAACCGTCAAGCAA | TCGGCGGCAATGAACAACT | 100,8 | 0,995 | Bariami et al. PLOS NTDs 2012 |
