# Supplementary material for: Insecticide resistance levels and mechanisms in Aedes aegypti populations in and around Ouagadougou, Burkina Faso
Source: PLoS Negl Trop Dis. 2019 May 23;13(5):e0007439. doi: 10.1371/journal.pntd.0007439 (PMC6550433; doi:10.1371/journal.pntd.0007439)
Supplement: S2 Table — (DOCX) [file pntd.0007439.s002.docx]

Table S2: 1H WHO Bioassay data

|  | |  | |  |  | |  |  |  |
| --- | --- | --- | --- | --- | --- | --- | --- | --- | --- |
| **Locality** | **Breeding site** | | **Insecticide** | | | **tested** | | **dead** | **alive** |
| Tabtenga | tyre | | Bendiocarb | | | 117 | | 117 | 0 |
| Tabtenga | tyre | | PBO+Permethrin | | | 80 | | 70 | 10 |
| Tabtenga | tyre | | Deltamethrin | | | 122 | | 61 | 61 |
| Tabtenga | tyre | | Permethrin | | | 97 | | 23 | 74 |
| Tabtenga | tyre | | PBO+deltamethrin | | | 88 | | 85 | 3 |
| Tabtenga | drum | | Deltamethrin | | | 139 | | 33 | 106 |
| Tabtenga | drum | | PBO+Deltamethrin | | | 80 | | 72 | 8 |
| Tabtenga | drum | | PBO+Permethrin | | | 120 | | 98 | 22 |
| Tabtenga | drum | | Permethrin | | | 128 | | 9 | 119 |
| Tabtenga | drum | | Bendiocarb | | | 78 | | 78 | 0 |
| Goundry | drum | | PBO+Permethrin | | | 40 | | 40 | 0 |
| Goundry | drum | | Deltamethrin | | | 94 | | 92 | 2 |
| Goundry | drum | | Permethrin | | | 105 | | 96 | 9 |
| Goundry | drum | | Permethrin | | | 103 | | 99 | 4 |
| Goundry | drum | | PBO+Permethrin | | | 91 | | 91 | 0 |
| Goundry | drum | | PBO+deltamethrin | | | 93 | | 93 | 0 |
| LG 1200 | tyre | | Deltamethrin | | | 85 | | 81 | 4 |
| LG 1200 | tyre | | Bendiocarb | | | 71 | | 71 | 0 |
| LG 1200 | tyre | | PBO+deltamethrin | | | 79 | | 79 | 0 |
| LG 1200 | tyre | | Deltamethrin | | | 95 | | 69 | 26 |
| LG 1200 | tyre | | PBO+Permethrin | | | 66 | | 64 | 2 |
| LG 1200 | tyre | | Permethrin | | | 76 | | 62 | 14 |
| LG 1200 | tyre | | Bendiocarb | | | 93 | | 92 | 1 |
|  |  | |  | | |  | |  |  |
