# Supplementary material for: Insecticide resistance levels and mechanisms in Aedes aegypti populations in and around Ouagadougou, Burkina Faso
Source: PLoS Negl Trop Dis. 2019 May 23;13(5):e0007439. doi: 10.1371/journal.pntd.0007439 (PMC6550433; doi:10.1371/journal.pntd.0007439)
Supplement: S4 Table — (DOCX) [file pntd.0007439.s004.docx]

**Supporting table 4: q-RT-PCR gene expression data.**

| **qPCR data** |  |  |  |  |  |  |  |  |  |  |  |
| --- | --- | --- | --- | --- | --- | --- | --- | --- | --- | --- | --- |
| Collection | samples | tech rep | 9J10 | 9J28 | 9M6 | 9J32 | 9J26 | RPS3 | Ae60s | 6BB2 | 6Z8 |
| 1200LG | 211-1 | 1 | 29,85 | 33,07 | 30,98 | 29,85 | 31,16 | 27,14 | 24,79 | 33,23 | 32,04 |
| 1200LG | 211-2 | 2 | 29,92 | 33,46 | 30,94 | 30,19 | 31,57 | 26,37 | 24,85 | 33,14 | 32,21 |
| 1200LG | 325-1 | 1 | 26,64 | 32,97 | 28,36 | 26,13 | 26,94 | 25,33 | 22,38 | 31,58 | 29,25 |
| 1200LG | 325-2 | 2 | 26,25 | 31,79 | 28,37 | 26,18 | 26,9 | 25,1 | 22,06 | 32,94 | 29,72 |
| 1200LG | 247-1 | 1 | 30,47 | 35,16 | 32,97 | 30,88 | 32,6 | 28,58 | 26,25 | 35,01 | 33,57 |
| 1200LG | 247-2 | 2 | 30,71 | 35,74 | 32,45 | 31,37 | 35,5 | 28,53 | 26,37 | 33,32 | 33,98 |
| 1200LG | 233-1 | 1 | 24,18 | 32,5 | 26,58 | 24,89 | 29,68 | 23,02 | 19,81 | 29,03 | 30,12 |
| 1200LG | 233-2 | 2 | 24,1 | 32,67 | 25,63 | 24,88 | No Ct | 22,8 | 20,42 | 29,1 | 30,17 |
| Tabtenga tire | 289-1 | 1 | 28,66 | 35,58 | 30,6 | 28,85 | 29,52 | 27,95 | 24,37 | 31,41 | 31,64 |
| Tabtenga tire | 289-2 | 2 | 28,27 | 33,93 | 30,3 | 29,93 | 29,33 | 27,37 | 24,53 | 31,46 | 31,66 |
| Tabtenga tire | 288-1 | 1 | 28 | 32,75 | 29,86 | 29,27 | 30,8 | 26,59 | 25,43 | 32,37 | 32,73 |
| Tabtenga tire | 288-2 | 2 | 27,51 | 32,28 | 29,9 | 29,67 | 30,61 | 26,32 | 24,65 | 32,07 | 31,65 |
| Tabtenga tire | 430-1 | 1 | 28,87 | 33,48 | 32,51 | 30,38 | 29,59 | 28,84 | 24,89 | 31,28 | 31,76 |
| Tabtenga tire | 430-2 | 2 | 28,67 | 33,43 | 31,82 | 29,68 | 29,8 | 28,04 | 24,56 | 30,77 | 32,28 |
| Tabtenga tire | 399-1 | 1 | 29,61 | 31,46 | 31,21 | 29,8 | 30,34 | 28,49 | 27,05 | 30,71 | 32,8 |
| Tabtenga tire | 399-2 | 2 | 29,2 | 31,87 | 31,46 | 29,93 | 29,53 | 28,93 | 26,68 | 30,86 | 32,66 |
| Tabtenga drum | 178-1-1 | 1 | 30,81 | 34,37 | 32,02 | 30,7 | 32,01 | 28,32 | 26,99 | 32,04 | 32,73 |
| Tabtenga drum | 178-1-2 | 2 | 31,06 | 34,22 | 31,62 | 30,51 | 31,51 | 29,18 | 26,87 | 32,72 | 31,31 |
| Tabtenga drum | 178-2-1 | 1 | 29,12 | 31,25 | 30,93 | 35,27 | 29,64 | 26,91 | 25,96 | 30,54 | 37,96 |
| Tabtenga drum | 178-2-2 | 2 | 28,81 | 31,08 | 30,45 | 29,75 | 31,45 | 26,95 | 25,88 | 30,74 | 31,91 |
| Tabtenga drum | 184-1 | 1 | 28,83 | 31,86 | 29,52 | 29,29 | 29,4 | 25,39 | 25,01 | 30,52 | 30,41 |
| Tabtenga drum | 184-2 | 2 | 28,67 | 32,19 | 29,44 | 29,04 | 29,44 | 25,99 | 24,53 | 29,88 | 30,49 |
| Tabtenga drum | 196-1 | 1 | 31,81 | No Ct | 32,92 | 32,29 | No Ct | 30,23 | 27,96 | 31,17 | 34,17 |
| Tabtenga drum | 196-2 | 2 | 32,73 | 37,23 | 33,9 | 31,89 | 34,87 | 30,31 | 27,94 | 31,44 | 34,84 |
| Goundry | 359-1 | 1 | 27,68 | 32,75 | 29,61 | 26,82 | 27,69 | 25,58 | 22,23 | 28,81 | 30,48 |
| Goundry | 359-2 | 2 | 27,25 | 32,56 | No Ct | 26,83 | 28,17 | 25,57 | 22,46 | 28,66 | 30,21 |
| Goundry | 356-1 | 1 | 28,17 | 34,13 | 30,12 | 27,82 | 29,1 | 24,81 | 21,89 | 28,96 | 30,25 |
| Goundry | 356-2 | 2 | 28,1 | 34,44 | 30,02 | 27,52 | 28,92 | 24,53 | 21,8 | 28,85 | 30,29 |
| Goundry | 337-1 | 1 | 29,4 | 36,5 | 29,9 | 30,52 | 31,16 | 26,41 | 24,33 | 29,89 | 31,6 |
| Goundry | 337-2 | 2 | 29,27 | 36,28 | 30,68 | 30,34 | 30,73 | 26,34 | 23,98 | 29,48 | 31,19 |
| Goundry | 350-1 | 1 | 31,38 | No Ct | 32,56 | 30,65 | No Ct | 28,51 | 25,95 | 30,68 | 33,29 |
| Goundry | 350-2 | 2 | 30,9 | 39,99 | 32,49 | 30,19 | 30,85 | 28,15 | 25,72 | 30,5 | 33,74 |
| Rockefeller | R1-1 | 1 | 24,29 | 29,42 | No Ct | 25,25 | 25,46 | 21,82 | 20,71 | 25,11 | 26,71 |
| Rockefeller | R1-2 | 2 | 24,36 | 29,16 | 25,82 | 25,62 | 25,54 | 22,23 | 20,23 | 25,18 | 26,82 |
| Rockefeller | R2-1 | 1 | 24,89 | 30,05 | 27,79 | 26,51 | 27,58 | 22,52 | 19,69 | 26,88 | 27,95 |
| Rockefeller | R2-2 | 2 | 24,68 | 29,94 | 27,8 | 26,5 | 27,29 | 22,65 | 20,13 | 27,3 | 27,97 |
| Rockefeller | R3-1 | 1 | 24,94 | 28,86 | 25,98 | 27,36 | 27,04 | 22,85 | 20,04 | 26,82 | 28,37 |
| Rockefeller | R3-2 | 2 | 24,93 | 29,97 | 25,98 | 26,8 | 26,49 | 22,75 | 20,12 | 26,14 | 28,16 |
