# Supplementary material for: Insecticide resistance levels and mechanisms in Aedes aegypti populations in and around Ouagadougou, Burkina Faso
Source: PLoS Negl Trop Dis. 2019 May 23;13(5):e0007439. doi: 10.1371/journal.pntd.0007439 (PMC6550433; doi:10.1371/journal.pntd.0007439)
Supplement: S5 Table — (DOCX) [file pntd.0007439.s005.docx]

**Supporting table 5. Genotyping Taqman data**

| **TaqMan data** | |  |  |  |  |
| --- | --- | --- | --- | --- | --- |
| Locality | S989P | V1016G | V1016I | F1534C | Haplotype |
| 1200LG | SS | VV | VV | FC | VF/VC |
| 1200LG | SS | 0 | VV | CC | VC |
| 1200LG | SS | VV | VV | FC | VF/VC |
| 1200LG | SS | VV | VV | CC | VC |
| 1200LG | SS | VV | VV | CC | VC |
| 1200LG | SS | VV | VV | CC | VC |
| 1200LG | SS | VV | VV | CC | VC |
| 1200LG | SS | VV | VV | CC | VC |
| 1200LG | SS | VV | VI | CC | VC/IC |
| 1200LG | SS | VV | VV | CC | VC |
| 1200LG | SS | VV | VI | CC | VC/IC |
| 1200LG | SS | 0 | II | CC | IC |
| 1200LG | SS | VV | VV | CC | VC |
| 1200LG | SS | VV | VV | CC | VC |
| 1200LG | SS | VV | VV | CC | VC |
| 1200LG | SS | VV | VI | CC | VC/IC |
| 1200LG | SS | VV | VV | CC | VC |
| 1200LG | SS | VV | VV | CC | VC |
| 1200LG | SS | VV | VI | CC | VC/IC |
| 1200LG | SS | VV | VV | CC | VC |
| 1200LG | SS | VV | VV | FC | VF/VC |
| 1200LG | SS | VV | VI | CC | VC/IC |
| 1200LG | SS | VV | VV | CC | VC |
| 1200LG | SS | VV | VV | CC | VC |
| Tabtenga | SS | VV | VI | CC | VC/IC |
| Tabtenga | SS | 0 | II | CC | IC |
| Tabtenga | SS | VV | VI | CC | VC/IC |
| Tabtenga | SS | VV | VI | CC | VC/IC |
| Tabtenga | SS | 0 | II | CC | IC |
| Tabtenga | SS | VV | VV | CC | VC |
| Tabtenga | SS | VV | VI | CC | VC/IC |
| Tabtenga | SS | VV | VV | CC | VC |
| 1200LG | SS | VV | VI | CC | VC/IC |
| 1200LG | SS | VV | VV | FC | VF/VC |
| 1200LG | SS | VV | VI | CC | VC/IC |
| 1200LG | SS | VV | VV | CC | VC |
| 1200LG | SS | VV | VV | FC | VF/VC |
| 1200LG | SS | VV | VV | CC | VC |
| 1200LG | SS | VV | VV | CC | VC |
| 1200LG | SS | VV | VI | FC | VI/FC* |
| 1200LG | SS | VV | VV | CC | VC |
| 1200LG | SS | VV | VV | CC | VC |
| 1200LG | SS | VV | VI | CC | VC/IC |
| 1200LG | SS | 0 | VI | CC | VC/IC |
| 1200LG | SS | 0 | VI | CC | VC/IC |
| 1200LG | SS | VV | 0 | CC | 0 |
| 1200LG | SS | VV | VI | CC | VC/IC |
| 1200LG | SS | 0 | II | CC | IC |
| 1200LG | SS | 0 | VI | CC | VC/IC |
| 1200LG | SS | VV | VI | CC | VC/IC |
| 1200LG | SS | 0 | II | CC | IC |
| 1200LG | SS | VV | VV | CC | VC |
| 1200LG | SS | VV | VV | CC | VC |
| 1200LG | SS | VV | VV | CC | VC |
| 1200LG | SS | VV | VI | CC | VC/IC |
| 1200LG | SS | VV | VV | CC | VC |
| Tabtenga | SS | VV | VV | CC | VC |
| Tabtenga | SS | VV | VV | CC | VC |
| Tabtenga | SS | VV | VI | CC | VC/IC |
| Tabtenga | SS | 0 | VV | CC | VC |
| Tabtenga | SS | VV | VV | FC | VF/VC |
| Tabtenga | SS | VV | VV | CC | VC |
| Tabtenga | SS | VV | VV | FC | VF/VC |
| Tabtenga | SS | VV | VV | CC | VC |
| Tabtenga | SS | VV | VV | CC | VC |
| Tabtenga | SS | 0 | II | CC | IC |
| Tabtenga | SS | VV | VV | CC | VC |
| Tabtenga | SS | VV | VV | CC | VC |
| Tabtenga | SS | VV | VV | CC | VC |
| Tabtenga | SS | VV | VI | CC | VC/IC |
| Tabtenga | SS | VV | VV | CC | VC |
| Tabtenga | SS | VV | VI | CC | VC/IC |
| Tabtenga | SS | VV | VV | FC | VF/VC |
| Tabtenga | SS | VV | VV | CC | VC |
| Tabtenga | SS | VV | VI | CC | VC/IC |
| Tabtenga | SS | VV | VV | CC | VC |
| Tabtenga | SS | VV | VV | FC | VF/VC |
| Tabtenga | SS | VV | VV | FC | VF/VC |
| Tabtenga | SS | VV | VI | CC | VC/IC |
| Tabtenga | SS | VV | VV | CC | VC |
| Tabtenga | SS | 0 | II | CC | IC |
| Tabtenga | SS | VV | VV | CC | VC |
| Tabtenga | SS | VV | VI | CC | VC/IC |
| Tabtenga | SS | VV | VI | CC | VC/IC |
| Tabtenga | SS | VV | VI | CC | VC/IC |
| Tabtenga | SS | VV | VI | CC | VC/IC |
| Tabtenga | SS | VV | VV | CC | VC |
| Tabtenga | SS | VV | VI | CC | VC/IC |
| Tabtenga | not tested | not tested | VV | CC | VC |
| Tabtenga | not tested | not tested | VV | CC | VC |
| Tabtenga | not tested | not tested | VV | CC | VC |
| Tabtenga | not tested | not tested | VV | CC | VC |
| Tabtenga | not tested | not tested | VV | CC | VC |
| Tabtenga | not tested | not tested | VI | CC | VC/IC |
| Tabtenga | not tested | not tested | VI | CC | VC/IC |
| Tabtenga | not tested | not tested | VI | CC | VC/IC |
| Tabtenga | not tested | not tested | VI | CC | VC/IC |
| Tabtenga | not tested | not tested | II | CC | IC |
| Tabtenga | not tested | not tested | VV | CC | VC |
| Tabtenga | not tested | not tested | VV | CC | VC |
| Tabtenga | not tested | not tested | VI | CC | VC/IC |
| Tabtenga | not tested | not tested | VI | CC | VC/IC |
| Tabtenga | not tested | not tested | VI | CC | VC/IC |
| Tabtenga | not tested | not tested | VV | CC | VC |
| Tabtenga | not tested | not tested | VI | CC | VC/IC |
| Tabtenga | not tested | not tested | VV | CC | VC |
| Tabtenga | not tested | not tested | VV | CC | VC |
| Tabtenga | not tested | not tested | VV | CC | VC |
| Tabtenga | not tested | not tested | VV | CC | VC |
| Tabtenga | not tested | not tested | VI | CC | VC/IC |
| Tabtenga | not tested | not tested | VV | CC | VC |
| Tabtenga | not tested | not tested | VI | CC | VC/IC |
| Tabtenga | not tested | not tested | VV | CC | VC |
| Tabtenga | not tested | not tested | II | CC | IC |
| Tabtenga | not tested | not tested | VV | CC | VC |
| Tabtenga | not tested | not tested | II | CC | IC |
| Tabtenga | not tested | not tested | VV | CC | VC |
| Tabtenga | not tested | not tested | VV | CC | VC |
| Tabtenga | not tested | not tested | VI | CC | VC/IC |
| Tabtenga | not tested | not tested | VV | CC | VC |
| Tabtenga | not tested | not tested | VV | CC | VC |
| Tabtenga | not tested | not tested | VI | CC | VC/IC |
| Tabtenga | not tested | not tested | VV | CC | VC |
| Goundry | not tested | not tested | VV | FC | VF/VC |
| Goundry | not tested | not tested | VV | FF | VF |
| Goundry | not tested | not tested | VV | FF | VF |
| Goundry | not tested | not tested | VV | FF | VF |
| Goundry | not tested | not tested | VV | FC | VF/VC |
| Goundry | not tested | not tested | VV | FF | VF |
| Goundry | not tested | not tested | VV | FF | VF |
| Goundry | not tested | not tested | VV | FF | VF |
| Goundry | not tested | not tested | VV | FC | VF/VC |
| Goundry | not tested | not tested | VV | FF | VF |
| Goundry | not tested | not tested | VV | FC | VF/VC |
| Goundry | not tested | not tested | VV | FF | VF |
| Goundry | not tested | not tested | VV | FF | VF |
| Goundry | not tested | not tested | VV | FC | VF/VC |
| Goundry | not tested | not tested | VI | CC | VC/IC |
| Goundry | not tested | not tested | VV | FF | VF |
| Goundry | not tested | not tested | VV | FC | VF/VC |
| Goundry | not tested | not tested | VV | FC | VF/VC |
| Goundry | not tested | not tested | VV | FF | VF |
| Goundry | not tested | not tested | VV | FC | VF/VC |
| Goundry | not tested | not tested | VV | FC | VF/VC |
| Goundry | not tested | not tested | VV | 0 | 0 |
| Goundry | not tested | not tested | VV | 0 | 0 |
| Goundry | not tested | not tested | VV | 0 | 0 |
| Goundry | not tested | not tested | VV | 0 | 0 |
| Goundry | not tested | not tested | VV | 0 | 0 |
| Goundry | not tested | not tested | VV | 0 | 0 |
| Goundry | not tested | not tested | VV | 0 | 0 |
| Goundry | not tested | not tested | VV | 0 | 0 |
| Goundry | not tested | not tested | VV | FF | VF |
| Goundry | not tested | not tested | VV | FF | VF |
| Goundry | not tested | not tested | VV | FC | VF/VC |
| Goundry | not tested | not tested | VV | FF | VF |
| Goundry | not tested | not tested | VI | CC | VC/IC |
| Goundry | not tested | not tested | VV | FC | VF/VC |
| Goundry | not tested | not tested | VV | FF | VF |
| Goundry | not tested | not tested | VV | FF | VF |
| Goundry | not tested | not tested | VV | FF | VF |
| Goundry | not tested | not tested | VV | FF | VF |
| Goundry | not tested | not tested | VI | FC | VI/FC* |
| Goundry | not tested | not tested | VV | FC | VF/VC |
| Goundry | not tested | not tested | VV | FC | VF/VC |
| Goundry | not tested | not tested | VV | 0 | 0 |
| Goundry | not tested | not tested | VV | FF | VF |
| Goundry | not tested | not tested | VV | CC | VC |
| Goundry | not tested | not tested | VV | FC | VF/VC |
| Goundry | not tested | not tested | VV | FC | VF/VC |
| Goundry | not tested | not tested | VV | FC | VF/VC |
| Goundry | not tested | not tested | VV | FC | VF/VC |
| Goundry | not tested | not tested | VV | FC | VF/VC |
| Goundry | not tested | not tested | VI | CC | VC/IC |
| Goundry | not tested | not tested | II | CC | IC |
| Goundry | not tested | not tested | VV | 0 | 0 |
